# Colloidal Analogues of Charged and Uncharged Polymer Chains with Tunable Stiffness**

**DOI:** 10.1002/anie.201202592

**Published:** 2012-08-15

**Authors:** Hanumantha Rao Vutukuri, Ahmet Faik Demirörs, Bo Peng, Peter D J van Oostrum, Arnout Imhof, Alfons van Blaaderen

**Affiliations:** Soft Condensed Matter, Debye Institute for Nanomaterials Science, Utrecht UniversityUtrecht (The Netherlands) E-mail: h.r.vutukuri@uu.nla.vanblaaderen@uu.nl

**Keywords:** anisotropic particles, chain structures, colloids, electric fields, polymers

Spherical colloids have been used successfully as condensed-matter model systems for studying fundamental aspects of both phase behavior^1^ and dynamic processes such as the glass transition.^2^ Recently, a growing interest in colloidal particles with more complex shapes and interactions is fueled by applications in self-assembly and advanced functional materials, as well as by the demand for more realistic colloidal model systems for molecular structures.^3^ Examples of such colloidal particles include rod-like particles,^4^ regular clusters,^5^ chiral chains,^6^ staggered chains,^7^ granular polymers,^8^ particles that exhibit inverse dipolar interactions,^9^ patchy particles,^10^ and step-growth polymerization of inorganic rod-like nanoparticles.^11^ The assembly of colloids into polymer-like chains would constitute a significant step forward in the design of colloidal analogues of molecular structures.3a

Polymers are typically divided according to stiffness into three different types, namely rigid rods, semiflexible polymers and flexible polymers.^12^ Biopolymers (semiflexible) such as microtubules, actin, and DNA have become useful systems for the study of fundamental aspects of polymer physics because they have several advantages over synthetic polymers.^13^ However, even for semiflexible biopolymers, current microscopy techniques cannot be used to study the dynamics of the chains on the individual monomer level.^13^, ^14^ Previous work on the preparation of rigid colloidal bead chains was based on using microfluidic trapping of particles in confined geometries in combination with thermal fusing of particles.^15^ It has previously been demonstrated that the stiffness of magnetic bead chains can be tuned by controlling the length of specific linker molecules between beads; this type of tuning has been done in the case of linkers based on the streptavidin—biotin interaction.^16^ This method relies on the nature of the particular functional group on the surface of the magnetic particles that binds to the linker molecule and involves several additional chemical steps.^16^ Additionally, magnetic materials usually have a high density and strongly absorb light, making them less suitable for the study of concentrated systems in real space.

Herein, we describe new methods to produce colloidal-particle chains of three stiffness regimes that can be observed on a single-particle level, that is, on the level of the monomers that make up the chain; the chains can even be observed in concentrated systems without using molecular tracers. These methods rely on the following: dipolar interactions induced by external electric fields in combination with long-range charge repulsion to assemble the particles into chains only, and a bonding step to ensure that the particles remain assembled as chains even after the external field is switched off. We can control the length and the flexibility of the chains. Additionally, we demonstrate that our method is generally applicable by using it to prepare several other colloidal polymers, such as block-copolymer chains, which are formed by combining rigid and flexible chains, spherocylinders, which are formed by heating rigid chains, and both atactic and isotactic chains, which are formed from heterodimeric-particle monomer units. We demonstrate that the flexibility of the charged chains can be tuned from very rigid (rod-like) to semiflexible (as in the simplified polymer model of beads on a string^17^) by changing the ionic strength. This method can, in principle, be used with any type of colloidal particle. Moreover, our systems can be matched in terms of refractive index and density, so that bulk measurements in real space are possible.

Colloidal particles that have dielectric constant values that are different from that of the solvent, acquire a dipole moment that is parallel to an external electric field. Dispersions of particles with induced dipolar interactions and with long-range screened Coulombic interactions show rich phase behavior.^18^ The dispersion structure can be tuned from chains (1D), to sheets (2D), and eventually to equilibrium 3D crystallites by varying the dipolar field strength.^18^ However, when the field is turned off, the dipolar interactions no longer exist and so the particles return to a dispersion.

Making use of this behavior in combination with either thermal annealing or seeded growth on inorganic, organic, and hybrid particles, we demonstrate that permanent bead chains can be made starting from different types of colloids.

Suspensions consisting of monodisperse polymethyl-methacrylate (PMMA) particles in cyclohexyl bromide (CHB) were introduced into a thin indium tin oxide (ITO) coated electric cell (Figure 1 j). Upon application of an external AC electric field, the induced dipole moment in each particle caused the particles to assemble into chains, one particle thick, aligned in the direction of the field in a head-to-tail arrangement and with a broad distribution in the length of the chains (*E*_rms_*=*0.20 V μm^−1^, *f*=1 MHz where *E*_rms_ is the root-mean-square electric-field strength and *f* is the frequency). In the low-field regime, the stable structure is a string fluid phase that consists of chains of particles parallel to the field direction and a liquid-like order of particles perpendicular to the field direction.^18^ The time scale for chain formation is on the order of a few seconds. In addition, by increasing the applied field strength, the average chain length could be increased until it spanned the entire width of the gap between the two electrodes. The long-range charge repulsions are essential because they greatly stabilize the individual chains, thus preventing them from forming sheets by sideways assembly at higher field strengths.18a Furthermore, the chains become straighter and stiffer upon increasing the strength of the dipole–dipole interactions, thus suppressing the thermal fluctuations. Using a hot-air stream that was much wider than the sample cell, the entire sample was heated to 70–75 °C, which is still well below the glass-transition temperature^19^ (*T_g_*=140–145 °C) of PMMA, and was allowed to remain at that temperature for about 2–3 min. After removing the field, it was found that more than 95 % of the dispersion was converted into permanent bead chains with a broad distribution in the length of the chains (Figure 1 a). At elevated temperatures, the steric PHSA–PMMA comb-graft stabilizer that is present on the surface of the particles, redistributes in such a way that the particles that are in contact become permanently fastened together by the same van-der-Waals forces between PMMA polymers that keep the uncrosslinked particles together. The chain structure remains intact even after the field is removed; the chains remained well dispersed owing to the long-range repulsion (*κ*σ≍0.8, where *σ* is the particle diameter (1.4 μm) and *κ* is the inverse of the Debye screening length, a measure for the range of the electrostatic repulsion). Even at higher salt concentrations the dispersion of chains was stable suggesting that the particles were still sterically stabilized.18a The length of the permanent PMMA chains was controlled by varying the distance between the two electrodes (Figure 1 a–c); in this way, more monodisperse chains of the desired length could be produced. We estimated that the polydispersity index for a dispersion of rigid bead chains, which are shown in Figure 1 c, is 1.11. Figure 1 e shows a scanning electron microscope (SEM) image of a single PMMA chain. In a concentrated dispersion of fluorescently labeled long bead chains, a nematic phase was observed in three dimensions by using confocal microscopy (Figure 1 d, see Movie S1 in the Supporting Information); using this technique, single particles in the chains could be clearly distinguished. Notably, the nematic phase possesses long-range orientational order, but no long-range positional order. Additionally, we were able to use the 3D information to calculate the orientational-order parameter, which was determined to be approximately 0.8, a value that is consistent with a nematic phase.^20^ The nematic-phase transition was accelerated by an external electric field.

**Figure 1:**
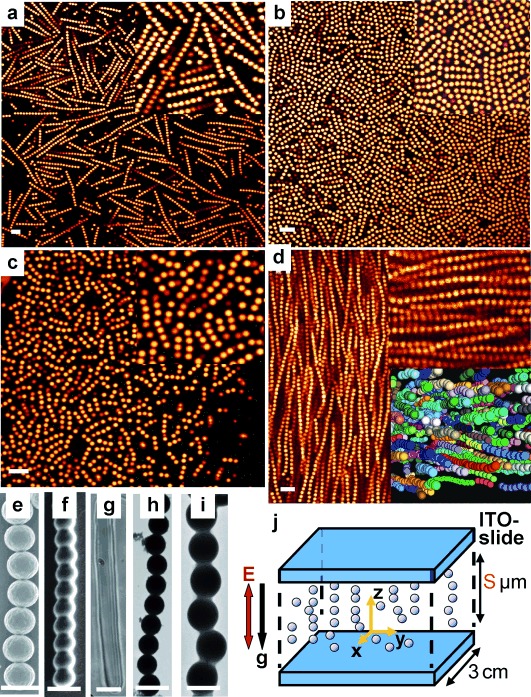
Permanent rigid colloidal bead chains. a–c) Confocal micrographs of permanent PMMA chains in CHB where the chain length was controlled by varying the distance (S) between the two electrodes; the distances were 100 μm, 10–15 μm, and 5–10 μm, respectively; the upper insets are magnified views of the bead chains. d) Confocal micrograph of the nematic phase of long bead chains of PMMA in CHB; upper inset is a magnified view of the micrograph, which has been rotated 90°; lower inset is a rendered representation of particle coordinates and reveals the three-dimensional structure. e–f) Scanning electron microscopy (SEM) images of permanent bead chains of PMMA and PS, respectively. g) Optical micrograph of a spherocylinder of PS, which was prepared by a prolonged heating of bead chains in DMSO. h–i) Transmission electron microscopy (TEM) images of permanent bead chains of silica (SiO_2_) and amorphous titania (TiO_2_), respectively. j) Schematic representation of the electric cell. Scale bars: a–d, 5 μm; e–h, 2 μm; i, 1 μm.

We used our method to make rigid bead chains consisting of different polymeric particles, such as polystyrene (PS) particles (Figure 1 f), and hybrid particles containing a silica core and a PMMA shell (see the Supporting Information). In addition, the shape of the PS bead chains could be changed by prolonged heating (4 h) at 95 °C; upon this treatment, the bead chains transformed into spherocylinders (Figure 1 g). A slightly modified procedure was used to produce permanent rigid inorganic colloidal bead chains as well. This modified procedure, which involved the growth of a thin layer of material around the chain, was used to prepare titania- (Figure 1 i) and silica-covered spheres (Figure 1 h; see the Supporting Information).

We made further modifications to our simple method to fabricate (semi)flexible chains of dielectric beads. PS bead chains in dimethyl sulfoxide (DMSO) became flexible when the PS beads were sterically stabilized with a higher molecular weight polyvinylpyrrolidone (PVP, *M*_W_≥360 kg mol^−1^; see Movie S2 in the Supporting Information). The longer bead chains exhibited different conformations and the interconversion between conformations was driven entirely by thermal fluctuations (Figure 2 a–d). We quantified the flexibility of the chains by estimating the persistence length (*l_p_*) of our rigid (approximately 40 mm or 30 000 *σ*) and semiflexible (approximately 14 μm or 10 *σ*, and the Kuhn length was approximately 20 *σ*; see the Supporting Information) particle chains using Fourier mode analysis.^21^ The ratio between the persistence length and the contour length (*l_p_*/*l_c_*) of our (semi)flexible chains is on the order of 1; this ratio is larger than 1000 for our rigid rods (see the Supporting Information, Figure S9). This value can be compared with the following: for flexible polymers such as λ-DNA, the ratio, *l*_p_/*l*_c_, is much less than one (*l*_p_≍50 nm, *l*_c_≍16 μm); for semiflexible polymers, such as actin filaments, the ratio is on the order of one (*l*_p_≍16 μm, *l*_c_≍20 μm);^21^ for rigid polymers such as microtubules, the ratio is on the order of 1200 (*l*_p_≍6 mm, *l*_c_≍50 μm). The distribution of bond angles was calculated for flexible and rigid 8-bead chains (Figure 2 e and f), a distribution that was determined as being Gaussian. By comparison of the bond-angle distribution with a Boltzmann distribution, it can be determined that the bond angle of 20 degrees has an elastic-energy cost of 1 *k*_B_ *T.* To further probe the flexibility, a single bead in a chain was trapped with optical tweezers and dragged through the dispersion. Hairpin-like conformations were observed and are a result of the viscous drag on the chain (see Movie S3 in the Supporting Information). In addition, ring- and knot-like structures could be made with a single optical trap on a long semiflexible chain (Figure 2 g–j). Once the optical trap was released, electrostatic repulsion, elastic energy, and entropy caused the chain to relax. Notably, when the PS particles were stabilized with a stabilizer of higher molecular weight, that is, PVP, the chains became flexible, whereas in the case of electrostatically stabilized particles, the chains stiffened (see the Supporting Information). Therefore, we believe that the stabilizing polymers (PVP) act as linkers between the beads by being entangled with the PS polymer chains.

**Figure 2:**
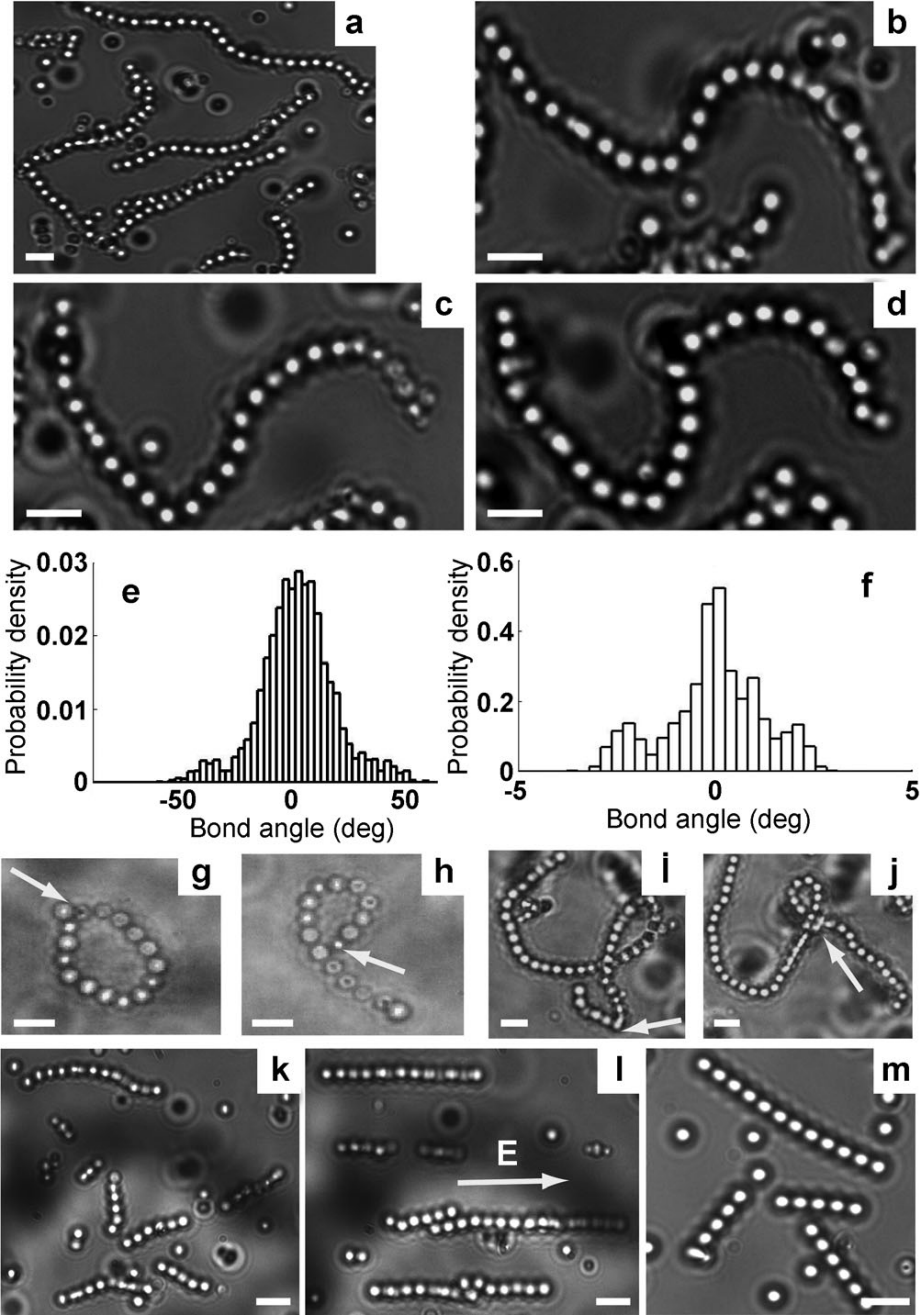
(Semi)flexible colloidal bead chains. a) Optical micrograph of PS bead chains in DMSO. b–d) Optical microscopy images of a long chain that is confined to the glass surface by gravity in DMSO taken at different times, a series of images that show the different conformations. e–f) The distribution of bond angles for a flexible and a rigid 8-bead chain, respectively. g–j) Optical micrographs of PS bead chains, in which a single bead in a chain was trapped with optical tweezers (indicated by an arrow) and dragged through the dispersion, thus forming a ring- and a knot-like structure. k–l) Conformations of PS chains in DMSO, in the absence and in the presence of an external field (*E*_*rm s*_=0.06 V μm^−1^, *f*=1 MHz), respectively. m) (Semi)flexible PS chains in deionized DMSO (*κσ*≍1, *σ*=1.35 μm). Scale bars: 4 μm.

More complex chains could be made by applying our method to a dispersion of PVP-stabilized heterodimeric colloids that consisted of PMMA (*σ*=1 μm, with a rough surface in the SEM inset of Figure 3 a and b) on one side and PS (*σ*=0.85 μm, with a smooth surface in the SEM inset of Figure 3 a and b) on the other side. Chains formed from these particles were also observed to be flexible. The dielectric constants of these two materials are similar (*ε*_PMMA_=2.6, *ε*_PS_=2.4), and thus the order of heterodimers within the chain was random, a behavior that is analogous to that of atactic polymers (Figure 3 a, Movie S4). However, control over the internal structure of a chain would be important in, for example, photonic applications. We therefore exploited the difference in charge between both sides of the heterodimer (PS–PMMA) to obtain control over the internal structure of the heterodimer chains. Prior to chain formation, the particles were first aligned in a particular direction by using a low DC field (1.5 V mm^−1^) for about 10–15 s; the side with the most negative zeta potential—in this case the PS side—points toward the positive electrode. The particles were then exposed to an AC field of 1 MHz for a much longer time (2–3 mins), and then heated. The resulting heterodimer chains were similar to isotactic polymers. The different refractive indices of both ends of the heterodimeric particles (1.59 for the PS end and 1.49 for PMMA end) allowed the ends to be distinguished using optical microscopy. The darker PS ends are distributed in a regular manner within the chain (Figure 3 b); this result is strikingly different from the random arrangement of heterodimeric particles in the previous experiment (lower inset of Figure 3 a; see also the Supporting Information, Figure S11). Additionally, an SEM image of the chain clearly shows that the smooth parts (PS) of the chain are regularly arranged (upper inset, Figure 3 b); this image can be compared with the SEM image of a chain in the previous experiment, in which the arrangement of smooth parts is random (upper inset, Figure 3 a).

**Figure 3:**
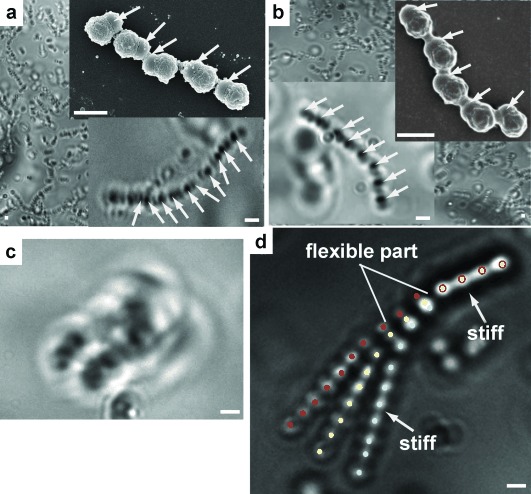
Complex chains. a–b) Optical micrographs of a permanent semiflexible atactic-like and isotactic-like chain of PMMA–PS heterodimers, respectively, in water; upper insets are SEM micrographs of the heterodimer chains; lower insets are magnified optical images of the heterodimer chains; the arrows indicate the smooth (PS) parts in the SEM images and the dark parts (PS) in the optical images (see the Supporting Information, Figure S11). c) Optical image of a single chain of heterodimers in the presence of depletion attractions. d) Color-coded overlay of optical micrographs of a triblock-copolymer-like chain of PS beads taken at different times; the overlay was constructed by placing the rigid end in the same position and orientation. Scale bars: 2 μm.

An interesting feature of these flexible chains is that the flexibility of already-synthesized charged chains can be tuned by manipulating the interactions between the beads in the chain. These interactions can be manipulated in three ways: by applying an external electric field (Figure 2 k–l), by varying the range of electrostatic repulsions (1/*κ*; Figure 2 m), and by inducing depletion attractions (Figure 3 a and c). The (semi)flexible PS bead chains became rigid when the chains were transferred from a normal solvent (*κσ*≍5, *σ*=1.35 μm; DMSO) to a strongly deionized solvent with *κσ*≍1, which is similar to that of polyelectrolytes^23^ (Figure 2 m, Movie S5). The stronger long-range electrostatic repulsions between the beads in such a solvent forced them into a straight conformation. Furthermore, we induced short-range attractions by adding a nonadsorbing polymer (1.75 wt % dextran of *M_w_*=5000 kg mol^−1^), which acted as a depletant to the semiflexible PS–PMMA heterodimer chains in water. The attractive depletion interactions between the beads caused the semiflexible chains to form a folded state (Figure 3 c, Movie S6). The conformational states of a polymer are highly dependent on the monomer–solvent and monomer–monomer interactions. The tuning of flexibility plays an important role in polymer physics. Although Doi predicted that rotational diffusion of a stiff polymer in a crowded environment is independent of stiffness,^22^ Odijk estimated that such diffusion should be enhanced by flexibility.^23^

We were also able to prepare chains that were flexible in one part and rigid in the other, a property that is characteristic of triblock copolymers (Figure 3 d and Movie S7). We prepared these chains by mixing flexible and rigid chains together and subjecting the mixture to the same protocol that was used in making the constituent bead chains.

In conclusion, we have developed a general method for preparing dielectric bead chains of high purity with a high yield. The method can be applied generally because it relies only on i) a repulsive double-layer interaction that is in the size range of the particles, ii) an induced dipolar attractive force in the direction of an external electric field and iii) short-range attractions that keep the particles together; such attractions include van der Waals forces, linkages formed using molecules such as PVP, and thin layers formed by seeded growth. We also showed that the semiflexible and rigid bead chains behave in a similar way to a variety of polymers and simplified polymer models.^24^ Altering the interactions between the colloidal beads, the interactions between bead and solvent, and the molecular weight of the linker (PVP) enables exquisite control over chain conformations and new insights into the behavior of simplified polymer models down to the monomer level in real space. Moreover, we have shown that our method can also be applied to particles of more complex shape and composition. We believe that the method could be applied to smaller particles by using the “giant” electrorheological effect.^25^

## Supplementary material

Detailed facts of importance to specialist readers are published as ”Supporting Information”. Such documents are peer-reviewed, but not copy-edited or typeset. They are made available as submitted by the authors.
