# Supplementary material for: Colloidal Analogues of Charged and Uncharged Polymer Chains with Tunable Stiffness
Source: Angew Chem Int Ed Engl. 2012 Aug 15;51(45):11249–53. doi: 10.1002/anie.201202592 (PMC3556699; doi:10.1002/anie.201202592)
Supplement: Supplementary file 8 [file anie0051-11249-SD8.pdf]

Angewandte Chemie
Eine Zeitschrift der Gesellschaft Deutscher Chemiker

Supporting Information

© Wiley-VCH 2012

69451 Weinheim, Germany

Colloidal Analogues of Charged and Uncharged Polymer Chains with Tunable Stiffness**

Hanumantha Rao Vutukuri, Ahmet Faik Demirörs, Bo Peng, Peter D. J. van Oostrum, Arnout Imhof, and Alfons van Blaaderen**

anie_201202592_sm_miscellaneous_information.pdf
anie_201202592_sm_vutukuri_movies1.mov
anie_201202592_sm_vutukuri_movies2.mov
anie_201202592_sm_vutukuri_movies3.mov
anie_201202592_sm_vutukuri_movies4.mov
anie_201202592_sm_vutukuri_movies5.mov
anie_201202592_sm_vutukuri_movies6.mov
anie_201202592_sm_vutukuri_movies7.mov

SUPPLEMENTARY MOVIE CAPTIONS

Movie S1: This movie is composed of a stack of confocal micrographs (XY-Z) of a nematic phase of stiff long PMMA bead chains in CHB. The focal plane is moved through the sample at 1.27 μm/s.

Movie S2: This movie shows different conformations of a (semi-)flexible PS chain in DMSO. The movie was acquired at 1 fps and it is played at 3 fps.

Movie S3: This movie illustrates the degree of flexibility of PVP stabilized PS bead chains. Hairpin, ring and knot like structures were made with a single optical trap on a long PS chain in DMSO. The movie was acquired at 20 fps and it is played at 40 fps.

Movie S4: This move shows a-tactic polymer like chains of dimer (PS-PMMA) particles in water. Note the irregular ordering of the dark (PS) and the bright (PMMA) parts in the chain. The movie was acquired at 1 fps and it is played at 6 fps.

Movie S5: This movie demonstrates the effect of the ionic strength on the flexibility of PVP-stabilized PS bead chains. PS chains were first in a normal DMSO and then transferred to strongly de-ionized DMSO. The movie was acquired at 3 fps and it is played at 15 fps.

Movie S6: This movie shows the depletion attraction driven folded state of a PMMA-PS dimer chain in water. The movie was acquired at 1 fps and it is played at 10 fps.

Movie S7: This movie shows a PS chain similar to a triblock copolymer that consisted of a 5-bead flexible middle part and stiff end parts of lengths 3 and 6, respectively. The movie was acquired at 1 fps and it is played at 8 fps.

Supplementary Information

Experimental Section

Materials

Particle synthesis: We used polymethylmethacrylate (PMMA), polystyrene (PS), and silica spherical particles. We synthesized PMMA particles by dispersion polymerization, covalently labelled them with the fluorescent dye 7-nitrobenzo-2-oxa-1, 3-diazol (NBD) or rhodamine isothiocyanate (RITC) and sterically stabilized with poly(12-hydroxystearic acid) grafted on a PMMA backbone. [1] We prepared silica particles using a modified Stöber procedure.[2] Sterically stabilized PS particles were made by dispersion polymerization.[3] The particle sizes were 0.6 µm, 1.4 µm, 2.04 µm for PMMA, 1.0 µm, 3.0 µm for silica and 1.35 µm, 1.54 µm for PS, as determined by static light scattering (SLS) and electron microscopy methods. Cyclohexyl bromide (CHB, Fluka), cis-decalin (Sigma-Aldrich), and polyvinylpyrrolidone (PVP, Sigma) were used as received. We used two types of sample cells, rectangular capillaries (VitroCom, UK) and home made sandwich ITO coated glass cells. The rectangular sample cell consisted of a 0.1 x 1.0 mm capillary with two 50 µm thickness nickel-alloy wires (Goodfellow) threaded along the side walls. We used a function generator (Agilent, Model 3312 OA) and a wide band voltage amplifier (Krohn-Hite, Model 7602M) to generate the electric fields.

Microscopy: After filling the cell with the colloidal suspension, we sealed it with UV-curing optical adhesive (Norland no.68), and we studied particle dynamics by means of confocal laser scanning microscopy (Leica TCS SP2) and bright field microscopy. After drying the sample, structures that were made permanent by thermal annealing were imaged with a scanning electron microscopy (FEI Phenom scanning electron microscopy).

Electrical conductivity measurements: DMSO was purified and ions were removed by contact with the mixed ionic resin beads (Bio-Rad AG 501-X8 Resin, 20-50 mesh, Biotechnology grade) for 4-5 days. We estimated the Debye screening length of our suspensions by measuring the conductivity of the normal and de-ionized DMSO (with a Radiometer analytical CDM 230 conductivity meter) and then applying Walden's rule.

Methods Summary

I. Permanent rigid chains of polymeric particles

a. Protocol for permanent rigid PMMA chains.

The dispersion was prepared at a volume fraction of φ = 0.04 from 1.40 µm RITC labelled PMMA particles in cyclohexyl bromide (CHB, Fluka). Such a dispersion was then transferred into an electric capillary cell of 0.05 mm X 1.0 mm (VitroCom, UK) cross section with a desired length and exposed to a strong AC field ($E_{rms} = 0.25$ *V/µm*, *f* = 1 *MHz* where E_{rms} is the root-mean-square electric field and *f* is the frequency) . After 5-6 minutes, nearly all of the particles were assembled into one particle wide strings in the field direction. The dispersion was then heated to 70-75 ºC, which is still well below the glass

transition temperature (T_g = 140-145 ºC) of PMMA, for about 2-3 minutes using a stream of hot air that was much wider than the sample cell. At elevated temperatures particles that were in contact bonded (or fused) irreversibly. We kept the field on for 5 minutes while the sample cell was allowed to cool down to room temperature. The field was then removed and confocal scanning laser microscopy revealed that the particles that had been in contact were bonded permanently (Fig. S1). Furthermore, we repeated the same procedure for different volume fractions and found that the upper workable limit is 10 % by volume. In this limit, the long-range repulsion still stabilized individual strings. [4] To probe the mechanical stability of bead chains, a 17 bead long stiff PMMA chain was trapped horizontally in an array of three time-shared optical traps. The two end particles of the chain were held in stationary traps, while the middle trap was moved up-down horizontally. Thanks to the entanglement of the polymers we didn't observe any breakage of the chain. This is likely to have stretched the bonds between the beads with several tens of k_bT so the absence of breakage suggests mechanical stability.

We adapted the same procedure to produce rigid chains of different polymeric particles namely hybrid particles such as silica (core)-PMMA (shell) (Fig. S2), and polystyrene (Fig. S3).

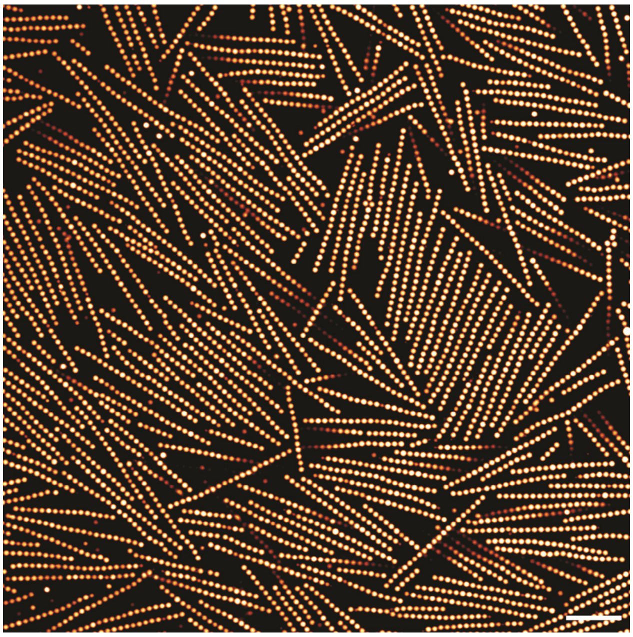

Fig. S1. Confocal microscopy image of 2D snapshot of permanent rigid PMMA chains in CHB. Scale bar, 5 µm.

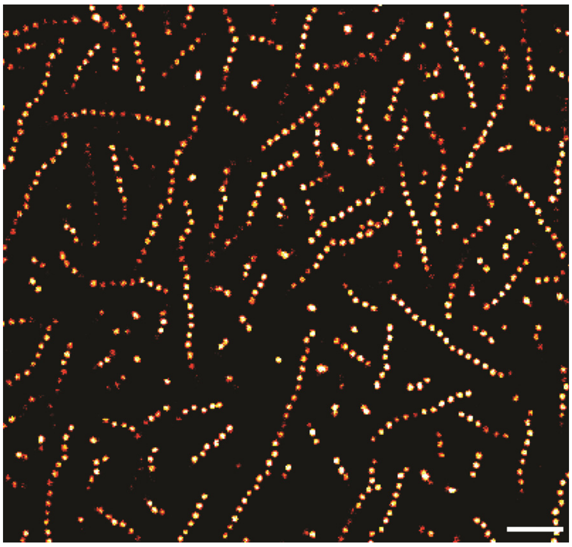

Fig. S2. Confocal microscopy image of 2D snapshot of permanent silica (core) –PMMA (shell) chains in CHB. Scale bar, 5 μm.

b. Protocol for permanent rigid PS chains.

The dispersion consisted of electrostatically stabilized PS particles[5] (diameter σ = 1.45 μm, 1 wt %) in dimethyl sulfoxide (DMSO, Fluka), and the dispersion was introduced by capillarity in a cell of 0.1 mm X 1.0 mm (VitroCom, UK) cross section with a desired length. Prior to the heating step, particles were aligned parallel to the field direction in head-to-toe manner due to induced dipolar attractions ($E_{rms} = 0.15$ *V/μm*, *f* = 1 *MHz)*. Such a sample was heated to 70-75 ºC, which is still well below the glass transition temperature[6] (T_g = 105 ºC) of PS, for about 2-3 minutes using a stream of hot air that was much wider than the sample cell. At elevated temperatures particles which were in contact bonded (or fused) irreversibly. Then the dispersion was allowed to cool down to room temperature.

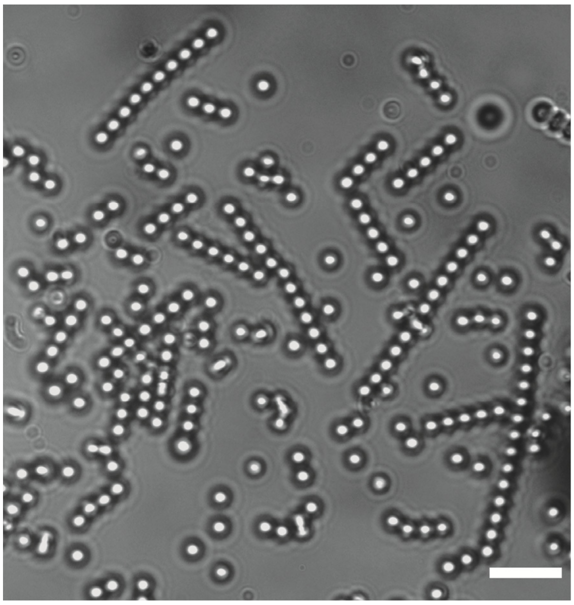

Fig. S3. Optical microscopy image of rigid PS chains in DMSO. Scale bar, 5 μm.

II . Permanent rigid chains of inorganic colloidal particles.

a. Protocol for a thin layer of silica coating around silica and titania chains.

1.3 μm silica particles were synthesized with a modified Stöber method.[2] A dispersion was prepared at 0.8 % by volume of particles in a 15% (v/v) ammonia (25 % aqueous solution, Merck) and glycerol (Sigma) mixture. A 10 mL portion of such a dispersion was mixed with 100 μL tetraethyl orthosilicate (TEOS, >98%, Fluka). Then, 5.0 μL of the final solution was transferred to an electric cell of 0.1 mm x 1.0 mm (VitroCom, UK) and exposed to an external AC field ($E_{rms} = 0.20$ V/μm, *f* = 1 *MHz*). Under these conditions, a thin layer of about 60-70 nm of silica was grown around chains which were aligned in the field direction with the help of dipolar attractions. Note that the reaction was done at room temperature and the reaction time was about 3-4 hrs. In this fashion both silica and amorphous titania[7] colloidal chains were fixed. The strings were dried on a glass slide and observed with TEM. If a thicker coating was desired, subsequent additions of TEOS were made (seeded growth). During the growth step the chemical reaction increases the ionic strength of the dispersion, which can hamper the colloidal stability of these chains.

b. Protocol for a reaction free method for permanent rigid silica chains in aqueous solution

The following is a reaction free method to produce rigid silica chains. Fluorescent – isothiocynate (FITC) labelled silica particles with an average diameter of 1.30 μm were prepared at 1.0 % by volume in a 0.3 % by weight of polyethyleneimine (PEI, M_w = 750 kg/mole, Sigma) DMSO solution. The suspension was allowed to stand for 3-4hrs. During this period the cationic PEI molecules adsorbed on the anionic surface of the particles. The solution was then introduced by capillarity in an electric cell of 0.1 mm X 1.0 mm (Vitro) cross section and subjected to a sufficiently strong AC field ($E_{rms} = 0.25$ *V/μm*, *f* = 1 *MHz*) for 20-30 minutes. Strong dipolar attractions aligned the silica particles and allowed the PEI molecules to irreversibly link them. After removing the field, the chains structure was preserved as shown in Fig. S4.

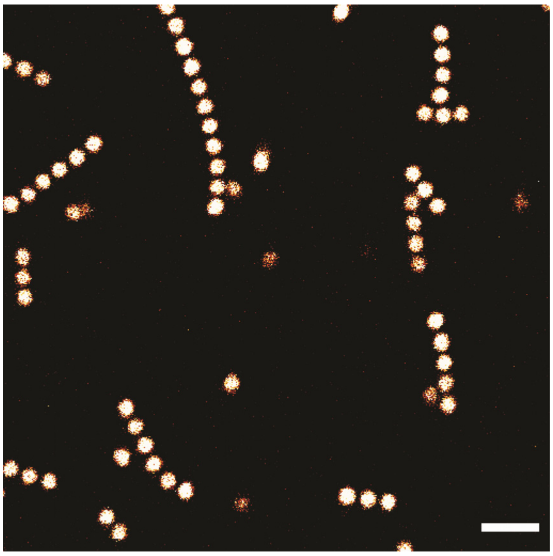

Fig. S4. Confocal microscopy image of 2D snapshot of permanent rigid silica chains in DMSO. Scale bar, 2 μm.

However, the stability of these systems is limited by the range of the repulsion (1/κ) and it is a challenge to get sufficiently long-range repulsions in aqueous suspensions. Hence to overcome this limitation, we synthesized silica chains in an apolar solvent (CHB) as well with a slightly modified method: First, a reaction was carried out between 3-methacryloxypropyltrimenthoxysilane (TPM, Sigma) and silica particles to stabilize the latter in CHB.

The TPM coating reaction was as follows: 0.34 g of 2.50 μm silica particles were dispersed in 15 mL of ethanol (Sigma), 1 mL of ammonia (25% aqueous solution, Merck) and 2 ml of TPM. The reaction mixture was allowed to react for 2 hrs. The particles were then washed a few times with ethanol and dried at room temperature. In a closed 200 ml glass laboratory flask, 15 mg of poly (12-hydroxystearic acid) and 5 g of azo-bis-isobutyronitrile (AIBN, Janssen Chemica) was dissolved in 10 g of CHB. Dried TPM coated particles were transferred to that solvent mixture. 5.0 μL of suspension was placed between the two electrodes then a reaction was carried out between TPM-coated particles in the presence of an AC field ($E_{rms} = 0.20$ *V/μm*, *f* = 1 *MHz*) at 70 ºC for 3-4 hrs. After the field was turned off, permanent rigid silica chains resulted (Fig. S5).

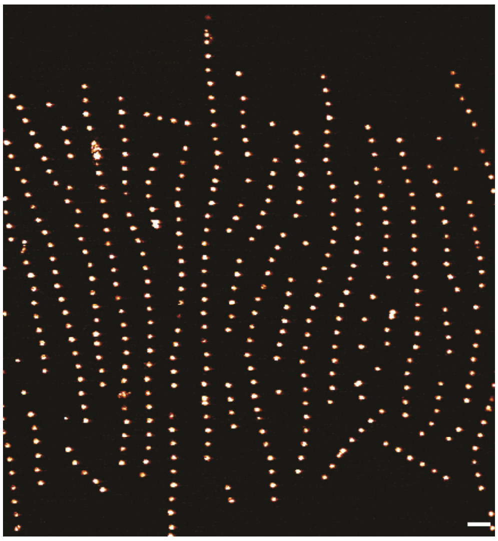

Fig. S5. Reflection mode confocal microscopy image of 2D snapshot of permanent rigid silica chains in CHB. Scale bar, 5 μm.

III. Flexible chains of polymeric particles.

a. Protocol for flexible PS chains.

i. Procedure for sterically stabilized PS particle synthesis

Materials

Styrene (St, Fluka) was passed over an aluminum oxide contained column to remove the inhibitor. After the inhibitor was removed, St was stored in a refrigerator. Azo-bis-isobutyronitrile (AIBN, Janssen Chemica) was re-crystallized in ethanol before use. Polyvinylpyrrolidone (PVP, Sigma) with average molecular weight 360,000 g/mol (K-90) was used as stabilizer. Dimethyl sulfoxide (DMSO, Fluka) and ethanol (Fisher) were used as supplied.

The standard recipe for post-addition dispersion polymerization of styrene[3] is listed in Table 1. The following procedure was used: All of the stabilizer (PVP), initiator (AIBN), one third of the monomer and half of the ethanol were added to a 250

ml three-necked flask equipped with a gas supply, a condenser, and a Teflon-coated stirring bar. After a homogeneous solution formed at room temperature, nitrogen was bubbled through the reaction system for 30 min. Then, the flask was immersed in a silicon oil bath, maintained at 70 ºC and stirred at 100 rpm. The remaining two thirds of the monomer was dissolved in the remaining ethanol at 70 ºC under nitrogen. Half of this hot mixture was added into the reaction flask after the polymerization reaction had run for 1 h, and the rest of the mixture was added after the polymerization reaction had run for 2 h. The reaction mixture was maintained at 70 º C for 24 h before cooling. The final particles suspension was washed 3 times with ethanol using a centrifuge to remove the free stabilizer. The obtained particles were dried under a nitrogen flow and stored at room temperature. The size of the particles was 1.54 μm as determined by static light scattering (SLS) and electron microscopy methods.

Table 1. Recipe for the polymerization of styrene in ethanol

Chemicals	Mass (g)
Styrene	15
PVP	1
Ethanol	50
AIBN	0.025

ii. Protocol for producing flexible PS chains.

PVP (M_w = 360 kg/mol) stabilized 1.54 μm PS particles were prepared at 0.80 % volume fraction in DMSO. The suspension was transferred to an electric cell of 0.1 mm x 1.0 mm cross section with a desired length and subjected to an AC electric field ($E_{rms} = 0.10$ *V/μm*, f = 1 *MHz*) . The entire sample was heated to 60-65 ºC for about 90-120 seconds and then the suspension was cooled down to room temperature. Roughly 1-2 minutes later the field was turned off. Note that we only observed flexible chains when the particles were stabilized with higher molecular weight PVP ($M_w \geq$ 360 kg/mol) (Supplementary Movie S4). Furthermore, lower molecular weights of 10 kg/mol, 40 kg/mol resulted in rigid chains.

b. Protocol for making flexible hetero dimers.

We synthesized hetero dimer colloids by dispersion polymerization[3], which consisted of PMMA (σ = 1 μm) on one side and PS (σ = 1 μm) on the other side, both were stabilized with PVP (M_w = 360 kg/mol). Subsequently, we implemented the same protocol to these particles.

c. Protocol for producing semi-flexible PMMA chains.

Here we used RITC labelled uncrosslinked core (σ = 2.0 μm)- crosslinked PMMA shell (100 μm) particles dispersed in CHB. We repeated the same protocol as in section I a. which resulted in semi-flexible chains (Fig. S6).

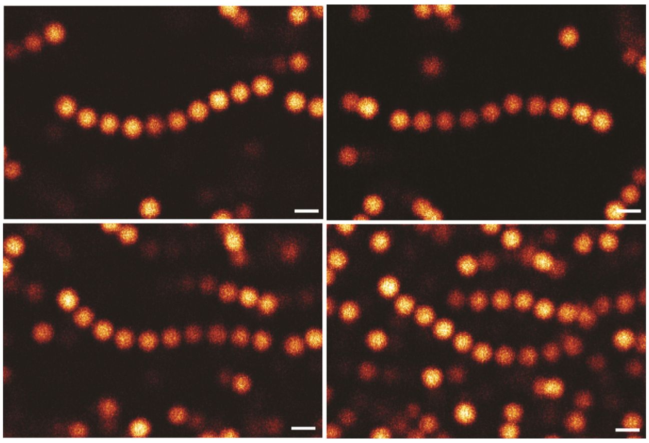

Fig. S6. Semi-flexible PMMA bead chain, Time-lapse confocal microscopy images show the different conformations. Scale bar, 3 μm.

IV. Numerical estimation of persistence length.

The persistence length is the distance along the chain over which the local orientation of the chain is no longer correlated. We estimated the persistence length of our flexible and rigid colloidal chains using Fourier mode analysis of their curvature change due to thermal fluctuations.[8] Note that our chains were constrained to a plane by gravity. We choose a chain which is isolated from neighboring chains to ensure negligible hydrodynamic coupling. We first processed recorded images and extracted the centroid position of each particle within the chain,[9] from the pixel coordinates (x_i, y_i) of the chain we calculated the tangent angle θ(*s*) as a function of arc length *s* using $\theta(s_i) = \tan^{-1}(y_{i+1} - y_i)/(x_{i+1} - x_i)$ where $s = \sum_{j=0}^{i+1} \sqrt{(x_{j+1} - x_j)^2 + (y_{j+1} - y_j)^2}$.

In Fourier mode analysis, the instantaneous curvature of a chain at a given time can be decomposed into a series of Fourier modes[8],

$$\theta(s) = \sum_{n=0}^{\infty} \theta_n(s) = \sqrt{\frac{2}{L}} \sum_{n=0}^{\infty} a_n \cos\left(\frac{n\pi s}{L}\right) \tag{1}$$

where *L* is the contour length of the chain, a_n is the amplitude of the nth mode. The time-averaged Fourier amplitude of each mode provides an independent estimate for the persistence length at its corresponding length scale. The bending energy *U* of a curved filament can be expressed as an integral of its deviation in curvature from the intrinsic curvature along the filament backbone[8]

$$U = \frac{1}{2}\kappa \int_0^L \left(\frac{\partial \theta}{\partial s} - \frac{\partial \theta_0}{\partial s} \right)^2 ds, \tag{2}$$

where $\kappa = k_B T$ x l_p is the bending rigidity, l_p is the persistence length, k_B is Boltzmann's constant, and T is the temperature. Furthermore, the bending energy can be expressed as a function of Fourier mode amplitudes by differentiation of eq.1 and subsequent integration along the arc length of the chain,

$$U = \frac{1}{2}\kappa \sum_{n=1}^{\infty} \left(\frac{n\pi}{L}\right)^2 \left(a_n - a_n^0\right)^2 \qquad (3)$$

Here, a_n^0 is the amplitude in the absence of applied or thermal forces and is non zero if the relaxed filament is not absolutely straight. In equilibrium, the equipartition theorem states that the average bending energy of each mode equals to ½ k_B T, so that from the eqs. 1 and 3 the persistence length can be written as [8]

$l_p = \dfrac{2}{\left(\dfrac{n\pi}{L}\right)^2 \operatorname{var}\left(a_n\right)}$, where $\operatorname{var}\left(a_n\right) = \langle \left(a_n\left(t + \Delta t\right) - a_n\left(t\right)\right)^2 \rangle_{t,\Delta t \square \tau}$, where Δt is the time lag between the different images, and τ is the relaxation time of the mode. We considered the first 3 modes and found that the persistence length l_p of an 8 bead flexible chain is ~14 µm (10σ, where σ is the particle diameter) with a standard deviation of 4.8 µm. On the other hand, the persistence length of a long rigid PS chain is ~40 mm (30000 σ). Note that rigid chains are too stiff to display significant fluctuations (Fig. S9). The ratio between the persistence length and the contour length (lp/lc) for our (semi-)flexible strings is on the order of 1, whereas this ratio is larger than 1000 for our rigid rods (Fig. S9). For a comparison, for flexible polymers such as λ-DNA this ratio is much smaller than one (lp ≈ 50 nm, lc ≈ 16 µm). In the case of semiflexible polymers such as actin filaments, the ratio is on the order of one (lp ≈ 16 µm, lc ≈ 20 µm). For rigid polymers such as microtubules the ratio is of the order of 1200 (lp ≈ 6 mm, lc ≈ 50 µm). Without any optimization we have already created flexible strings with a length of 100 beads and rigid strings of 70-80 particles in length. We believe that the contour length of our semi-flexible chains can easily be extended to a few hundreds of particle lengths. Therefore, our PS bead chains can be considered as flexible polymer analogues.

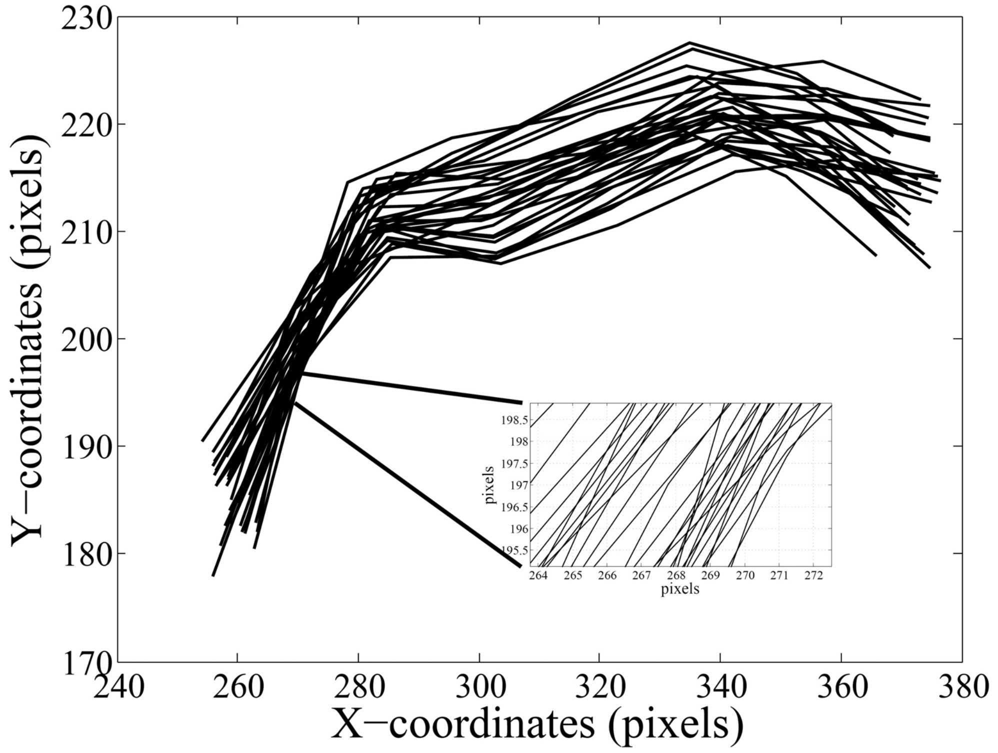

Fig. S7. The digitized (x, y) coordinates of 8 bead flexible PS chain in DMSO, inset, higher-magnification views of 30 consecutive chain contours from a video acquisition, which demonstrates that the filament tracking algorithm performs with subpixel accuracy.

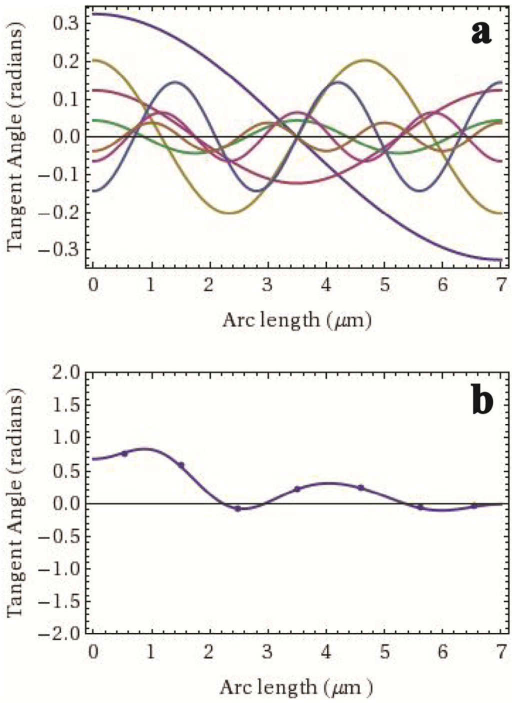

Fig. S8. a, Curves showing the 7 cosine modes into which the tangent-angle curve was decomposed. **b**, sum of the 7 modes was shown as a solid curve.

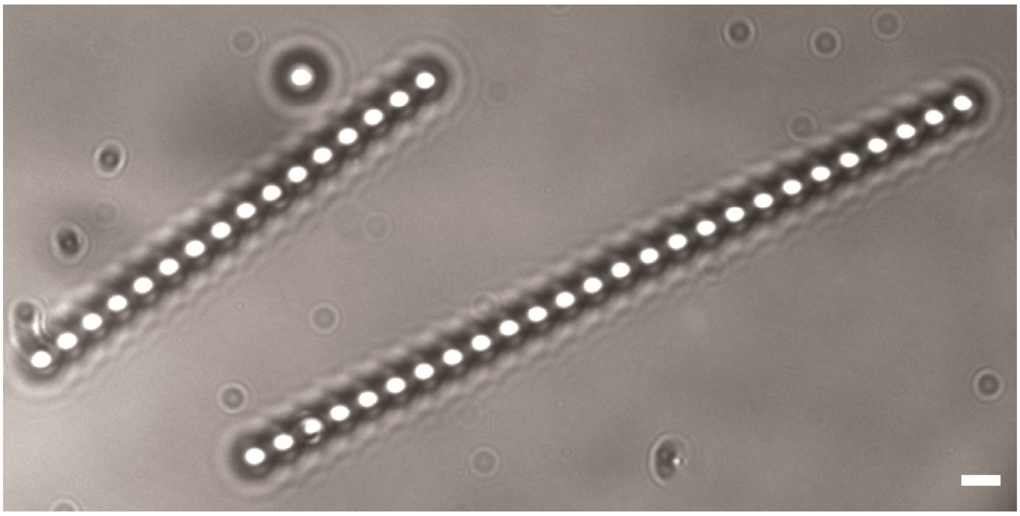

Fig. S9. Optical micrograph of a long rigid PS chains in DMSO. Scale bar, 2 μm.

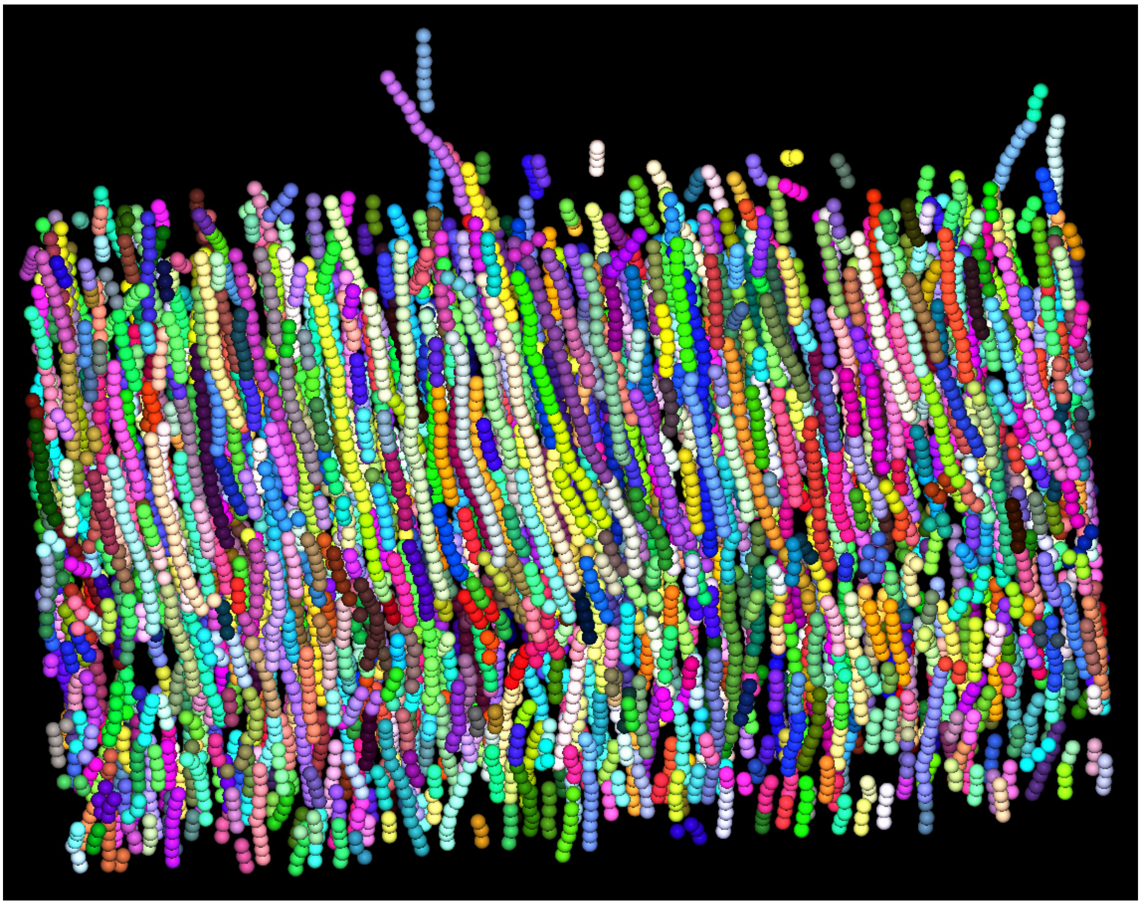

Fig. S10. Rendered particle coordinates revealing the three dimensional structure of a nematic phase of long bead chains.

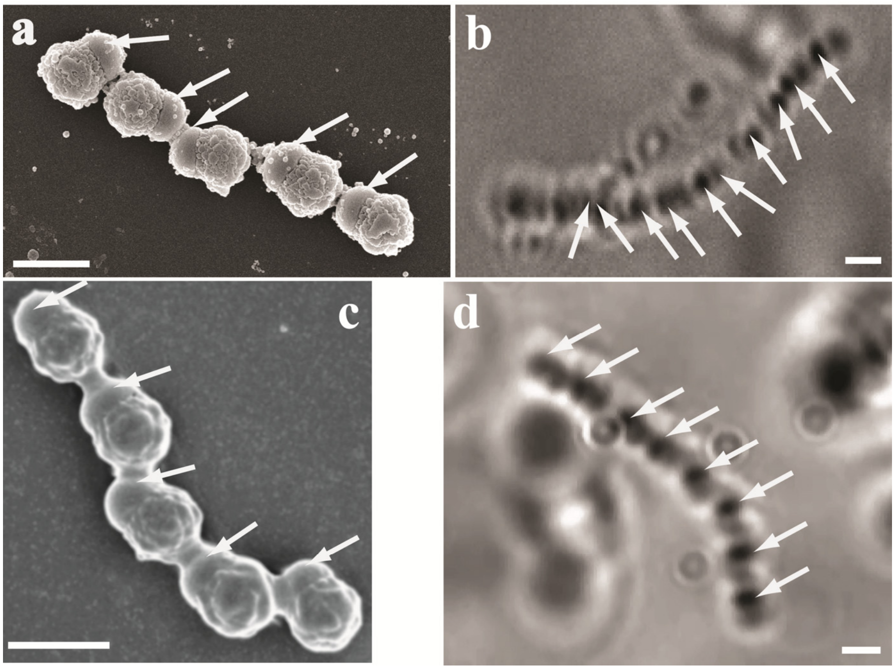

Fig. S11. Complex strings. a&d, Optical micrograph of a permanent semi-flexible chain of hetero dimers of PMMA-PS in water. a&b, SEM and optical micrographs of atactic like dimer chains respectively, and c&d, SEM and optical micrographs of isotactic like dimer chains respectively. We note that arrows indicate the smooth (PS) parts in SEM images, whereas the dark parts (PS) in optical images. Scale bars, 2 µm.

Supplementary references:

[1] G. Bosma, C. Pathmamanoharan, E. H. A. de Hoog, W. K. Kegel, A. van Blaaderen, H. N. W. Lekkerkerker, J. Colloid Interface Sci. **2002**, 245, 292-300.

[2] a) W. Stöber, A. Fink, E. Bohn, J. Colloid Interface Sci. **1968**, 26, 62-69; b) A. van Blaaderen, A. Vrij, Langmuir **1992**, 8, 2921-2931.

[3] a) J.-W. Kim, R. J. Larsen, D. A. Weitz, J. Am. Chem. Soc. **2006**, 128, 14374-14377; b) J.-S. Song, F. Tronc, M. A. Winnik, J. Am. Chem. Soc. **2004**, 126, 6562-6563.

[4] a) A.-P. Hynninen, M. Dijkstra, Phys. Rev. E **2005**, 72, 051402; b) A. Yethiraj, A. van Blaaderen, Nature **2003**, 421, 513-517.

[5] J. W. Goodwin, J. Hearn, C. C. Ho, R. H. Ottewill, Colloid Polym. Sci. **1974**, 252, 464-471.

[6] S. Mazur, R. Beckerbauer, J. Buckholz, Langmuir **1997**, 13, 4287-4294.

[7] S. Eiden-Assmann, J. Widoniak, G. Maret, J. Dispersion Sci. Technol. **2004**, 25, 535-545.

[8] a) C. P. Brangwynne, G. H. Koenderink, E. Barry, Z. Dogic, F. C. MacKintosh, D. A. Weitz, Biophys. J. **2007**, 93, 346-359; b) F. Gittes, B. Mickey, J. Nettleton, J. Howard, Journal of cell biology **1993**, 120, 923-934; c) A. Ott, M. Magnasco, A. Simon, A. Libchaber, Phys. Rev. E **1993**, 48, R1642-R1645.

[9] S. S. Rogers, T. A. Waigh, X. B. Zhao, J. R. Lu, Phys Biol **2007**, 4, 220-227.
